# Ultrasound Evidence of Fibrillar and Thickness Changes in Plantar Fasciosis Following 20% Dextrose Prolotherapy: Perifascial vs. Intrafascial Injection During a 1-Year Follow-Up

**DOI:** 10.3390/life16071051

**Published:** 2026-06-24

**Authors:** Alvaro Saura-Sempere, Ruben Sanchez-Gomez, Ismael Ortuño-Soriano, Ignacio Zaragoza-García, Paola Sanz Wozniak, José Manuel Reguera-Medina, Marta Martín-Vega, Alvaro Gómez-Carrión

**Affiliations:** 1 Nursing Department, Faculty of Nursing, Physiotherapy, and Podiatry, Universidad Complutense de Madrid, 28040 Madrid, Spain; alvarosaura@gmail.com (A.S.-S.); iortunos@ucm.es (I.O.-S.); izaragoz@ucm.es (I.Z.-G.); martma51@ucm.es (M.M.-V.); 2 Podiatry Clinic Álvaro Saura, Mairena del Aljarafe, 41927 Sevilla, Spain; 3 Instituto de Investigación Sanitaria Hospital Clínico San Carlos (IdISSC), 28040 Madrid, Spain; 4 Instituto de Investigación Sanitaria imas12, Grupo Invecuid, 28041 Madrid, Spain; 5 Faculty of Health Sciences, University of Castilla-La Mancha, 45600 Talavera de la Reina (Toledo), Spain; paolsanz@ucm.es; 6 Podiatry Clinic Sanipie, Utrera, 41704 Sevilla, Spain; josemaremed22@gmail.com; 7 Department of Nursing and Podiatry, Faculty of Health Sciences, University of Malaga, 29071 Malaga, Spain; alvaroalcore@hotmail.com

**Keywords:** plantar fasciosis, dextrose prolotherapy, ultrasound imaging, fibrillar pattern, tissue remodeling, chronic heel pain

## Abstract

Introduction: Plantar fasciosis is a common degenerative condition characterized by structural alterations of the plantar aponeurosis, including increased thickness and loss of its normal fibrillar pattern. Ultrasound imaging is widely used to assess these changes; however, the validity of plantar aponeurosis thickness as a marker of tissue recovery remains controversial. Dextrose prolotherapy has been proposed as an effective treatment for this condition, although its effects are not yet fully established. In particular, different injection approaches have been described, such as perifascial and intrafascial techniques, whose comparative effectiveness remains unclear. Therefore, the aim of this study was to evaluate longitudinal changes in plantar aponeurosis thickness and fibrillar pattern following treatment with these two prolotherapy approaches. Methods: A total of 56 patients with plantar fasciosis were prospectively evaluated over a one-year period following treatment with 20% dextrose prolotherapy. Ultrasound assessments were performed at multiple time points to measure plantar aponeurosis thickness and to qualitatively evaluate the fibrillar pattern (recovered vs. non-recovered). Longitudinal changes in thickness were analyzed using repeated-measures generalized linear models (GLMs), while differences in fibrillar pattern recovery according to injection technique (intrafascial vs. perifascial) were assessed using the chi-square test and Fisher’s exact test. Results: No significant changes in plantar aponeurosis thickness were observed over the follow-up period (*p* = 0.260), despite a slight decreasing trend. In contrast, recovery of the fibrillar pattern was observed in 92.9% of patients (52/56) at one year. The recovery rate was significantly higher in the perifascial group (100%) compared to the intrafascial group (80%) (Fisher’s exact test, *p* = 0.013). Qualitative fibrillar pattern assessment was independently re-evaluated by a blinded second examiner, demonstrating almost perfect inter-rater agreement (Cohen’s κ = 0.83). Conclusions: Dextrose prolotherapy was associated with ultrasonographic evidence of structural tissue remodeling of the plantar aponeurosis, as evidenced by recovery of the fibrillar pattern, without inducing significant changes in thickness. These findings suggest that assessment of the fibrillar pattern may offer additional information regarding structural remodeling following 20% dextrose prolotherapy, whereas plantar aponeurosis thickness remained relatively stable throughout follow-up.

## 1. Introduction

The plantar aponeurosis (PA) is a fundamental structure in foot biomechanics, extending from the medial calcaneal tuberosity to the proximal phalanges, and establishing connections with ligamentous and muscular structures that contribute to the stability of the plantar arch [1,2]. In addition, the PA forms part of a continuous myofascial system that includes the Achilles tendon, the crural fascia, and the posterior chain, integrating into the so-called achilles–calcaneal–plantar system [3,4,5,6]. This structural continuity enables efficient force transmission along the lower limb and plays a key role in locomotion [7].

Moreover, its mechanical properties vary regionally, being stiffer in its proximal portion and more compliant in distal regions, which has relevant diagnostic and therapeutic implications [8,9].

From a histological perspective, the PA exhibits a complex organization comprising longitudinally arranged type I collagen fibers, together with transverse and oblique fibers that confer resistance and adaptability to mechanical loading, as well as elastic fibers, hyaluronan, and rich sensory innervation [10]. The distinction between core and sheath provides a novel understanding of its mechanical and biological behavior, highlighting the greater regenerative capacity of the sheath compared with the predominantly structural role of the core [11]. Under pathological conditions, such as plantar fasciosis, significant structural alterations occur, including fibrillar disorganization, fibrosis, irregular angiogenesis, and accumulation of mucopolysaccharides [12].

Musculoskeletal ultrasound has become the imaging modality of choice for evaluating the plantar fascia due to its accessibility, dynamic capability, and high resolution [13,14,15]. The most characteristic sonographic findings include thickening, decreased echogenicity, irregular margins, and loss of the normal fibrillar pattern [16].

Traditionally, PA thickness has been considered a key marker in the assessment of plantar fasciosis. However, current evidence shows conflicting results: while some studies have reported a correlation between thickness reduction and structural or symptomatic evolution [17,18], others have found no significant changes despite symptomatic relief [19]. This variability calls into question its value as an isolated indicator of structural tissue remodeling.

From this perspective, the analysis of the fibrillar pattern by ultrasound becomes particularly relevant. Loss of fiber parallelism and the presence of hypoechoic areas reflect structural disorganization, whereas restoration of a homogeneous fibrillar pattern may indicate structural tissue remodeling processes. Furthermore, certain morphological features, such as fascial biconvexity, defined as a plantar aponeurosis with convexity of both superficial and deep borders, in contrast to a flatter morphology with more parallel fascial borders, have been identified as predictors of poor response to conservative treatment [20].

Therefore, the combined assessment of thickness and fibrillar pattern may provide a more comprehensive view of the structural status of the PA and its evolution over time, potentially allowing better correlation with patient-reported outcomes in future studies.

Among emerging regenerative therapies, dextrose prolotherapy has gained interest as a minimally invasive technique aimed at stimulating tissue repair through local inflammatory and proliferative mechanisms. Previous studies have reported symptomatic improvement in patients with plantar fasciosis treated with prolotherapy [21]; however, its structural effects on the PA—particularly in terms of thickness and fibrillar organization assessed by ultrasound—remain insufficiently characterized. In addition, the site of injection may influence the therapeutic response. Intrafascial injection would act directly on the degenerated structural tissue, whereas perifascial injection may preferentially stimulate the sheath, which has greater biological regenerative potential. Nevertheless, the current literature lacks conclusive evidence comparing both ultrasound-guided approaches [22,23,24,25,26].

The aim of this study was to evaluate longitudinal changes in plantar aponeurosis thickness and fibrillar pattern using ultrasound in patients with chronic plantar fasciosis treated with 20% dextrose prolotherapy under two different injection techniques (perifascial vs. intrafascial) over a one-year follow-up period, in order to determine their utility as ultrasonographic structural markers of tissue remodeling.

## 2. Materials and Methods

### 2.1. Study Design, Setting, and Ethical Considerations

This study was designed as a prospective, two-arm, randomized comparative study with a 52-week follow-up period. The study compared two ultrasound-guided injection techniques for 20% dextrose prolotherapy in patients with plantar fasciosis: intrafascial injection and perifascial injection.

The study was conducted in a specialized podiatry clinical setting between January 2025 and March 2026. Patients were recruited consecutively from individuals attending a specialized podiatry clinic for persistent plantar heel pain compatible with plantar fasciosis. Eligibility was assessed through clinical examination and diagnostic ultrasound prior to inclusion.

The study protocol was approved by the Ethics Committee of Hospital Clínico San Carlos, Madrid, Spain (reference C.I. 25/022-EC_X_Tesis), and the study was registered under ISRCTN29902542. All procedures were conducted in accordance with the ethical principles of the Declaration of Helsinki. Written informed consent was obtained from all participants before enrolment. Participants were informed that two ultrasound-guided injection approaches were being compared; however, they were not allowed to choose the injection technique.

### 2.2. Participants

Patients were eligible for inclusion if they met all of the following criteria: (1) age between 30 and 45 years; (2) clinical symptoms compatible with plantar fasciosis for at least 30 days; (3) ultrasound evidence of plantar aponeurosis involvement, defined as plantar aponeurosis thickness greater than 0.4 cm at the calcaneal insertion; and (4) provision of written informed consent.

Patients were excluded if they presented any of the following criteria: (1) systemic disease or morphofunctional alteration unrelated to the foot that could affect lower-limb biomechanics; (2) previous medical, orthotic, pharmacological, or invasive treatment involving the affected foot within the previous three months; (3) plantar heel pain attributable to other conditions, including autoimmune disease, nerve entrapment, traumatic injury, or other local pathology; or (4) inability to understand or comply with the study protocol.

A total of 62 patients were assessed for eligibility. Six patients were excluded: four did not meet the inclusion criteria and two declined to participate. Therefore, 56 participants were finally included and randomized.

### 2.3. Sample Size Calculations

Sample size estimation was performed before participant recruitment within a repeated-measures framework based on the primary ultrasound outcome, namely longitudinal changes in plantar aponeurosis thickness. Assuming a statistical power of 80%, a two-sided significance level of 0.05, and a moderate expected effect size, the minimum required sample size was estimated at 32 participants. To account for potential treatment discontinuations and improve the precision of longitudinal analyses, additional participants were recruited, resulting in a final sample of 56 patients.

### 2.4. Randomization and Allocation

Randomization was performed using a computer-generated allocation sequence before treatment initiation. No blocking or stratification procedures were applied. Participants were assigned to either the intrafascial or perifascial treatment group according to the pre-established randomization sequence.

Due to the single-center nature of the study, no formal allocation concealment procedure was implemented. However, participants were not allowed to choose the injection technique, and treatment allocation was determined exclusively by the randomization process.

Due to the nature of the ultrasound-guided intervention, blinding of the clinician performing the procedure was not feasible. Participants were not informed of the specific injection plane used during the intervention.

### 2.5. Ultrasound Equipment and General Assessment Protocol

Ultrasound examinations and ultrasound-guided procedures were performed using a General Electric LOGIQ e R8.0.9 system with software version 9.2 (GE Medical Systems-America, Milwaukee, WI, USA), equipped with a high-frequency linear transducer operating at 8–13 MHz.

All ultrasound examinations were performed with the patient in the prone position and the foot relaxed, with the ankle maintained in a neutral position as far as possible. The plantar aponeurosis was assessed at its calcaneal insertion, with particular attention to the area of greatest structural abnormality. Both longitudinal and transverse ultrasound views were obtained.

Plantar aponeurosis thickness was measured at the thickest point near the calcaneal insertion, using a standardized longitudinal view. Measurements were recorded in centimetres. Baseline images were stored for comparison with follow-up examinations.

Ultrasound follow-up assessments were performed at baseline and at 2, 4, 6, 8, 12, 26, and 52 weeks after treatment initiation.

### 2.6. Definition of Plantar Aponeurosis Morphology

Baseline plantar aponeurosis morphology was classified as either flat or biconvex according to its ultrasound appearance in the short-axis and longitudinal views. Flat morphology was defined as a plantar aponeurosis showing relatively parallel superficial and deep borders, without marked convexity of the fascial body. Biconvex morphology was defined as a plantar aponeurosis showing convexity of both the superficial and deep borders, producing a rounded or lens-shaped ultrasound appearance of the fascial body.

**Figure 1:**
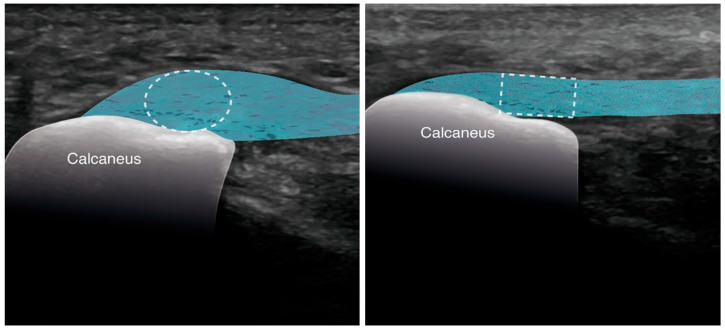
Schematic representation of the two plantar aponeurosis morphologies evaluated in the present study in the longitudinal plane. The image on the left shows a biconvex plantar aponeurosis, whereas the image on the right shows a flat plantar aponeurosis.

Classification was performed at baseline using stored ultrasound images. When the morphology was not clearly classifiable in a single image plane, both longitudinal and transverse views were reviewed. If the examiner considered that the morphology could not be confidently assigned to either category, the case was reviewed again using the complete baseline image set. No case was excluded on this basis because all participants could be assigned to one of the two predefined morphological categories after complete image review. Representative ultrasound images of flat and biconvex plantar aponeurosis morphology are provided in Figure 1.

### 2.7. Definition of Fibrillar Pattern and Fibrillar Pattern Recovery

The normal fibrillar pattern of the plantar aponeurosis was defined as a predominantly homogeneous echotexture with parallel hyperechoic fibrillar lines corresponding to the longitudinal collagen fibre organization.

An altered fibrillar pattern was defined as loss of the normal parallel fibrillar architecture, associated with hypoechoic areas, heterogeneous echotexture, fibre disorganization, or disruption of the normal linear echogenic structure.

Fibrillar pattern recovery was defined as restoration of a predominantly parallel hyperechoic fibrillar architecture at 52 weeks compared with baseline examination, together with disappearance or substantial reduction in the previously identified hypoechoic and disorganized areas.

The outcome was classified dichotomously as “recovered” or “not recovered”. A case was classified as recovered only when both longitudinal and transverse ultrasound views showed clear restoration of the fibrillar architecture compared with baseline images. A case was classified as not recovered when persistent hypoechoic disorganization, absence of clear fibrillar restoration, or relevant architectural disruption remained visible at 52 weeks.

Ambiguous cases were handled conservatively. If the examiner could not confidently identify clear restoration of the fibrillar pattern after comparison with baseline images, the case was classified as not recovered.

### 2.8. Examiner Assessment and Reliability

The primary ultrasound examination and the ultrasound-guided treatment procedures were performed by an experienced clinician with more than 10 years of experience in musculoskeletal ultrasound. Because the primary examiner performed the intervention, blinding to treatment allocation during the procedure was not feasible.

To reduce assessment bias, all stored baseline and 52-week ultrasound images were independently reviewed by a second examiner with experience in musculoskeletal ultrasound. The second examiner was blinded to treatment allocation, clinical evolution, chronological order of the images, and the initial examiner’s classification.

Fibrillar pattern recovery was classified independently by both examiners as recovered or not recovered. Interobserver agreement was assessed using Cohen’s kappa coefficient. Interpretation of kappa values followed the classification proposed by Landis and Koch.

### 2.9. Intervention Protocol

All patients received ultrasound-guided 20% dextrose prolotherapy under sterile conditions. The injectate consisted of 2 mL of 20% glucose solution combined with 0.2 mL of mepivacaine. The injection was administered using sterile syringes and fine-gauge needles suitable for ultrasound-guided musculoskeletal procedures.

Before each injection, ultrasound examination was performed to identify the area of greatest structural involvement at the calcaneal insertion of the plantar aponeurosis.

**Figure 2:**
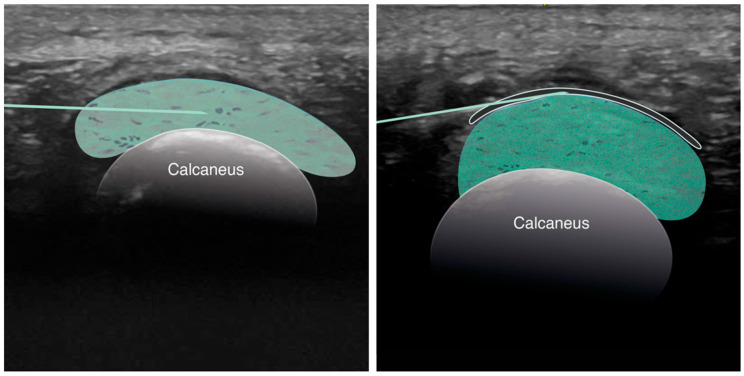
Representative ultrasound images illustrating the two prolotherapy injection techniques evaluated in the present study in the short-axis plane. The image on the left shows the intrafascial injection technique, characterized by retention of the injectate within the plantar aponeurosis, with variable distribution influenced by the degree of fascial degeneration. The image on the right shows the perifascial injection technique, characterized by deposition and spread of the injectate within the perifascial plane between the plantar aponeurosis and the overlying subcutaneous tissue.

In the intrafascial group, the needle was advanced under real-time ultrasound guidance into the thickness of the plantar aponeurosis, and the injectate was deposited within the fascial tissue. In the perifascial group, the needle was advanced under real-time ultrasound guidance to the interface between the plantar aponeurosis and the overlying subcutaneous tissue. The injectate was deposited within this perifascial plane, with ultrasound confirmation of spread along the interface (Figure 2).

All procedures were performed under real-time ultrasound visualization to confirm needle position and injectate distribution. Standard post-procedure recommendations were provided to all participants.

The number of treatment sessions was individualized according to clinical progression, with participants receiving repeated sessions during the treatment period when clinically indicated. Participants typically received between three and five treatment sessions according to clinical evolution. The total number of infiltrations was recorded for each patient and included in the descriptive analysis.

### 2.10. Follow-Up and Drop-Out Handling

Participants were followed for 52 weeks after treatment initiation. Ultrasound assessments were scheduled at 2, 4, 6, 8, 12, 26, and 52 weeks.

Treatment discontinuation and reasons for withdrawal were recorded prospectively. Participants who discontinued the treatment protocol but attended the final 52-week ultrasound assessment were retained in the final analysis. Therefore, the final analysis included all 56 randomized participants who underwent the 52-week ultrasound evaluation.

### 2.11. Outcome Variables

The primary outcome was the longitudinal change in plantar aponeurosis thickness, measured in centimetres at baseline and at each follow-up time point.

The secondary outcome was fibrillar pattern recovery at 52 weeks, classified as recovered or not recovered according to the predefined ultrasound criteria.

Additional variables included sex, body mass index, symptom duration, number of infiltrations, baseline plantar aponeurosis morphology, and treatment group.

### 2.12. Statistical Analysis

Continuous variables were expressed as mean ± standard deviation and 95% confidence intervals when appropriate. Categorical variables were expressed as absolute frequencies and percentages.

Baseline demographic, clinical, and ultrasound characteristics were reported for the full sample and separately for the intrafascial and perifascial groups. Between-group comparisons were performed using the independent-samples *t*-test or Mann–Whitney U test for continuous variables, depending on data distribution, and the chi-square test or Fisher’s exact test for categorical variables.

Normality of continuous variables was assessed using the Shapiro–Wilk test.

Longitudinal changes in plantar aponeurosis thickness were analyzed using a repeated-measures general linear model (GLM), with time as the within-subject factor and treatment group as the between-subject factor. Body mass index, symptom duration, and total number of infiltrations were included as covariates in the model. The group × time interaction was assessed to determine whether thickness evolution differed between intrafascial and perifascial approaches. Mauchly’s test was used to assess sphericity. When the sphericity assumption was violated, Greenhouse–Geisser correction was applied. Effect sizes were expressed as partial eta squared (η^2^*p*).

Fibrillar pattern recovery at 52 weeks was analyzed as a dichotomous categorical variable. Differences between treatment groups were assessed using contingency tables. The chi-square test was applied when assumptions were met, and Fisher’s exact test was used when expected cell counts were low.

Interobserver agreement for fibrillar pattern recovery was assessed using Cohen’s kappa coefficient.

Statistical significance was set at *p* < 0.05. All statistical analyses were performed using IBM SPSS Statistics, version 26.0 (IBM Corp., Armonk, NY, USA).

## 3. Results

### 3.1. Participant Flow and Follow-Up

**Figure 3:**
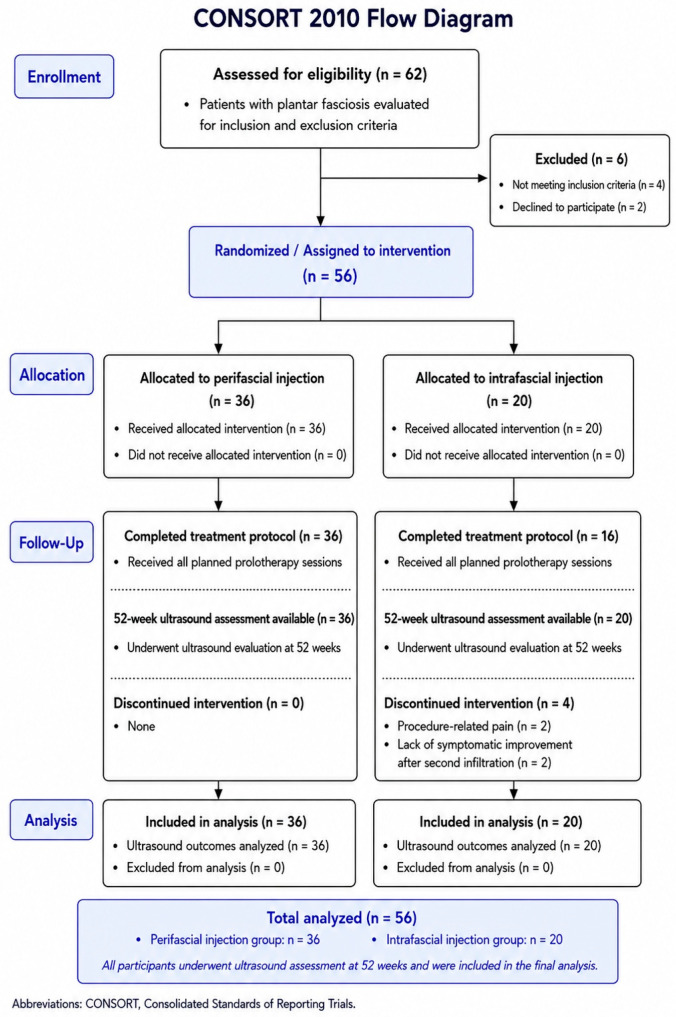
CONSORT 2010 flow diagram showing participant enrollment, randomization, follow-up, and analysis. All randomized participants completed the 52-week ultrasound evaluation and were included in the final analysis.

Participant flow throughout the study is summarized in Figure 3. Of the 62 patients assessed for eligibility, six were excluded, resulting in a final sample of 56 randomized participants.

All participants underwent the 52-week ultrasound follow-up assessment and were therefore included in the final analysis. Four participants allocated to the intrafascial treatment group discontinued the treatment protocol before completing all scheduled prolotherapy sessions. Two participants withdrew after the first infiltration because of severe post-procedural pain, and two discontinued treatment after the second infiltration because of a perceived lack of clinical improvement. No treatment discontinuations occurred in the perifascial group. Although these participants did not complete the planned treatment protocol, all attended the final 52-week ultrasound assessment and were retained in the final analysis.

### 3.2. Baseline Characteristics

Baseline demographic, clinical, and ultrasound characteristics according to treatment group are presented in Table 1.

### 3.3. Longitudinal Changes in Plantar Aponeurosis Thickness

The longitudinal analysis of plantar aponeurosis thickness by ultrasound did not demonstrate statistically significant changes throughout the 52-week follow-up period (Table 2). Mean values remained relatively stable from baseline (0.620 ± 0.113 cm) to 52 weeks (0.556 ± 0.189 cm), showing only a slight downward trend over time.

Repeated-measures general linear model analysis revealed no statistically significant longitudinal effect of time on plantar aponeurosis thickness (Greenhouse–Geisser corrected: F = 1.365; *p* = 0.260; partial η^2^ = 0.027). Although a progressive reduction in mean thickness was observed from weeks 6–8 onwards, these changes did not reach statistical significance.

When analyzed according to treatment group, baseline thickness values were similar between intrafascial and perifascial approaches (0.639 ± 0.110 cm versus 0.610 ± 0.114 cm, respectively). No significant group × time interaction was identified (Greenhouse–Geisser corrected: F = 2.351; *p* = 0.093; partial η^2^ = 0.046), indicating that the temporal evolution of plantar aponeurosis thickness did not differ significantly between injection techniques.

Overall, these findings suggest that plantar aponeurosis thickness remained relatively stable following prolotherapy throughout the analyzed follow-up period.

### 3.4. Fibrillar Pattern Recovery

**Figure 4:**
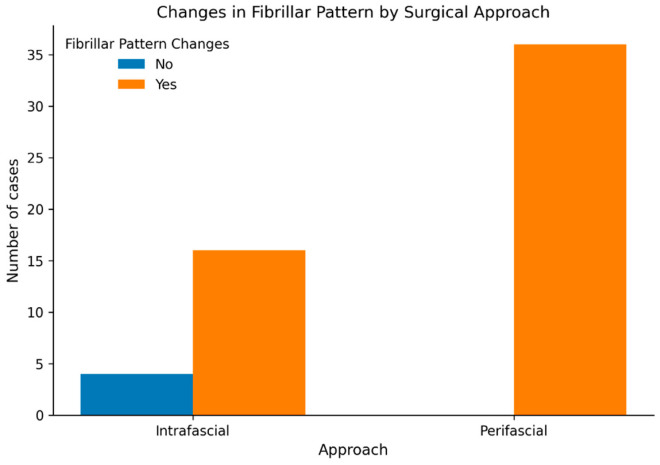
Distribution of fibrillar pattern recovery according to treatment technique. The perifascial group showed complete recovery (100%), whereas the intrafascial group presented a lower recovery rate (80%) and included all non-recovered cases.

The analysis of fibrillar pattern recovery demonstrated a high rate of structural reorganization at the end of the follow-up period (Table 3, Figure 4). Of the 56 participants included in the study, 52 (92.9%) showed recovery of the fibrillar pattern, whereas four participants (7.1%) exhibited persistent ultrasonographic abnormalities at 52 weeks.

When results were analyzed according to treatment technique, statistically significant differences were observed between groups. In the perifascial injection group, all participants (36/36; 100%) demonstrated fibrillar pattern recovery. In contrast, recovery was observed in 16 of 20 participants (80.0%) in the intrafascial group, whereas four participants (20.0%) showed no evidence of structural recovery.

These differences were statistically significant according to Fisher’s exact test (*p* = 0.013). Chi-square analysis yielded consistent results (χ^2^ = 7.754; *p* = 0.005), with continuity correction remaining statistically significant (*p* = 0.025).

**Figure 5:**
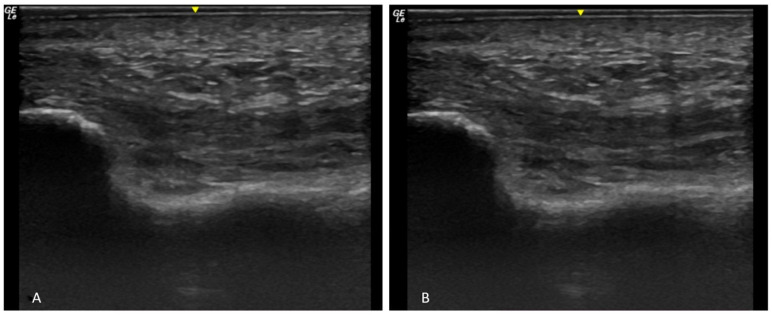
Representative longitudinal ultrasound images of a participant classified as recovered according to the predefined fibrillar pattern criteria. (**A**) Baseline ultrasound image showing disruption of the normal fibrillar architecture and diffuse hypoechogenicity in a plantar aponeurosis with flat morphology. (**B**) Ultrasound image obtained at the 52-week follow-up demonstrating recovery of the fibrillar pattern compared with baseline. According to the predefined ultrasound criteria, this case was classified as recovered.

**Figure 6:**
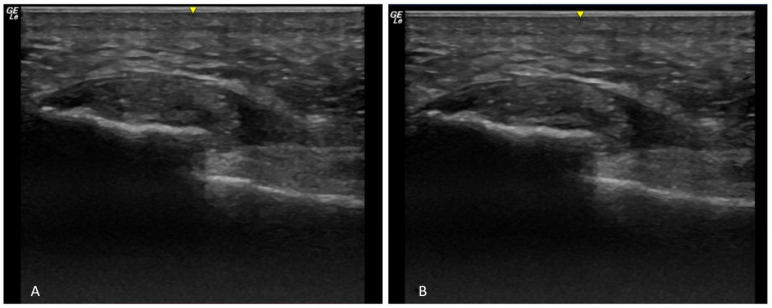
Representative longitudinal ultrasound images of a participant classified as not recovered according to the predefined fibrillar pattern criteria. (**A**) Baseline ultrasound image showing marked disruption of the normal fibrillar architecture and diffuse hypoechogenicity in a plantar aponeurosis with biconvex morphology. (**B**) Ultrasound image obtained at the 52-week follow-up demonstrating persistent loss of fibrillar organization compared with baseline. According to the predefined ultrasound criteria, this case was classified as not recovered.

Notably, all non-recovered cases belonged to participants allocated to the intrafascial treatment group. Representative ultrasound images of altered baseline fibrillar pattern, recovered fibrillar pattern, and persistent non-recovered fibrillar pattern are provided in Figure 5 and Figure 6.

### 3.5. Interobserver Agreement

Interobserver agreement for ultrasound assessment of fibrillar pattern recovery was evaluated in all participants included in the final analysis (n = 56). Baseline and 52-week ultrasound images were independently reviewed by the primary examiner and a second musculoskeletal ultrasound examiner blinded to treatment allocation, clinical outcomes, and the initial assessment.

Fibrillar pattern recovery was classified dichotomously as recovered or not recovered according to the predefined ultrasound criteria. Agreement between observers was quantified using Cohen’s kappa coefficient.

The overall observed agreement was 94.6% (53/56 cases), corresponding to a Cohen’s kappa coefficient of κ = 0.83 (Table 4), indicating almost perfect interobserver agreement.

## 4. Discussion

The aim of the present study was to evaluate longitudinal changes in plantar aponeurosis (PA) thickness and fibrillar pattern following treatment with 20% dextrose prolotherapy in patients with chronic plantar fasciosis. The main findings reveal a clear dissociation between morphometric and structural outcomes: while no significant changes were observed in PA thickness throughout the 12-month follow-up, a high proportion of patients (92.9%) demonstrated ultrasonographic recovery of the fibrillar pattern. These results call into question the validity of fascial thickness as an isolated marker of structural tissue remodeling.

(1) Reduction in PA thickness

Most previous studies evaluating plantar fasciosis treatments have primarily focused on plantar aponeurosis thickness as the main structural outcome measure. Corticosteroid injections have consistently been associated with short-term reductions in fascial thickness, particularly during the first 12 weeks following treatment [27,28,29,30]. Comparative studies evaluating different therapeutic modalities likewise report heterogeneous but generally progressive decreases in thickness over time [17,22,31,32,33,34,35,36]. Similarly, extracorporeal shockwave therapy and platelet-rich plasma interventions have also demonstrated progressive morphometric reductions during follow-up [37,38,39,40,41,42]. Other therapeutic approaches, including manual physiotherapy, ultrasound-guided procedures, and radiofrequency techniques, have likewise been associated with reductions in fascial thickness [41,42,43,44,45].

Despite these previously reported findings, the present study demonstrated no significant longitudinal changes in plantar aponeurosis thickness following prolotherapy, either in early or long-term follow-up phases. Although a slight decreasing trend was observed from weeks 6–8 onward, the magnitude of change remained limited and did not reach statistical significance. Furthermore, no significant group × time interaction was observed between intrafascial and perifascial approaches, suggesting that both techniques produced comparable morphometric trajectories.

In this context, particularly relevant are the findings of Gurcay et al. [22], who compared superficial and deep injection approaches and reported reductions in fascial thickness regardless of the injection plane. In contrast, the present study did not identify significant morphometric differences between intrafascial and perifascial prolotherapy approaches throughout follow-up, reinforcing the possibility that prolotherapy-induced structural responses may not be adequately reflected by thickness measurements alone.

Similarly, the randomized clinical trial by Karakılıç et al. [19] did not identify significant reductions in fascial thickness following prolotherapy despite symptomatic improvement reported in the original study. This finding is especially relevant because it closely parallels the dissociation observed in the present study between morphometric stability and ultrasonographic fibrillar pattern recovery. Comparable inconsistencies and heterogeneous longitudinal responses have also been described in studies evaluating extracorporeal shockwave therapy and regenerative interventions for plantar fasciosis [34,35]. Collectively, these findings suggest that plantar aponeurosis thickness may represent a relatively nonspecific structural parameter that does not necessarily reflect underlying tissue remodeling processes.

From a pathophysiological perspective, these findings may be explained by the fact that thickness reduction may occur secondary to multiple mechanisms, including transient reduction in edema, local inflammatory modulation, or mechanical unloading, rather than true collagen reorganization [12]. Consequently, quantitative morphometric changes alone may provide limited information regarding the biological quality of tissue repair. In this context, the results of the present study support the hypothesis that plantar aponeurosis thickness has limited utility as an isolated structural biomarker for monitoring tissue remodeling following prolotherapy.

(2) Changes in the fibrillar pattern

In contrast to the findings related to PA thickness, the present study demonstrated a high rate of fibrillar pattern recovery following prolotherapy. Loss of the normal fibrillar architecture is one of the characteristic ultrasonographic findings in plantar fasciosis [16], reflecting collagen disorganization and structural degeneration of the plantar aponeurosis [12]. Consequently, restoration of the fibrillar pattern may represent a more direct indicator of tissue remodeling than thickness measurements alone.

Most previous ultrasound studies in plantar fasciosis have predominantly focused on quantitative variables, particularly PA thickness. However, this approach may insufficiently characterize the true structural organization of the tissue. Even in studies evaluating regenerative approaches such as prolotherapy or platelet-rich plasma, structural assessment has largely relied on morphometric measurements rather than direct analysis of fibrillar organization. In contrast, the present study specifically evaluated qualitative fibrillar pattern restoration, allowing assessment of collagen reorganization beyond simple morphometric measurements.

The high proportion of patients demonstrating fibrillar recovery (92.9%) suggests that prolotherapy may be associated with structural remodeling processes characterized by restoration of fascial architecture. Nevertheless, the qualitative nature of the fibrillar pattern assessment should be interpreted cautiously until standardized ultrasonographic classification systems are developed and externally validated. Although interobserver agreement in the present study demonstrated almost perfect interobserver agreement (κ = 0.83), future investigations should incorporate semiquantitative or quantitative grading systems in order to improve reproducibility and reduce potential subjectivity.

Some previous studies have reported qualitative ultrasonographic changes following treatment, including modifications in echogenicity and fascial morphology after manual physiotherapy interventions [43]. However, unlike these studies, the present work specifically evaluated recovery of the fibrillar pattern, providing a more direct measure of structural tissue reorganization.

Furthermore, the absence of thickness changes reported in studies such as Karakılıç et al. [19], despite favorable symptomatic evolution reported by those authors, suggests that quantitative parameters may lack sufficient sensitivity. Our findings extend this observation by demonstrating that structural remodeling may occur in association with recovery of the fibrillar pattern, even in the absence of measurable changes in thickness.

Notably, all non-recovered cases belonged to participants allocated to the intrafascial treatment group. Although the plantar aponeurosis contains anatomically distinct compartments, the absence of significant differences in fascial thickness progression between intrafascial and perifascial injections may indicate that the biological effects induced by dextrose are not strictly confined to the initial deposition site. The rapid diffusion of hyperosmolar dextrose through interfascial and perivascular compartments could potentially expose both the fascial core and surrounding sheath to similar tissue-remodeling stimuli. Nevertheless, the higher proportion of fibrillar pattern recovery observed after perifascial prolotherapy may suggest that the perifascial sheath contributes more actively to tissue remodeling. Previous anatomical studies have described this region as a biologically active compartment with greater vascularity, higher cellularity, and increased regenerative potential compared with the dense collagenous core of the plantar aponeurosis [11].

An additional and clinically relevant observation was that all treatment discontinuations occurred in the intrafascial group, including two patients who reported severe pain after the first infiltration. Although the number of adverse-event-related withdrawals was limited, this finding may suggest that the perifascial approach is better tolerated than the intrafascial technique. One possible explanation is that intrafascial injection produces greater direct mechanical stimulation of degenerative tissue, potentially increasing post-procedural pain and local inflammatory response. However, these findings should be interpreted cautiously given the relatively small number of withdrawals and the absence of a specific tolerability analysis. Future studies with larger samples should further investigate the safety and tolerability profiles of both prolotherapy approaches.

Overall, the present findings support the concept that qualitative ultrasonographic parameters may provide more relevant information regarding tissue remodeling processes than isolated morphometric measurements. Consequently, the ultrasonographic fibrillar pattern may represent a more sensitive indicator of structural remodeling following prolotherapy, with potential utility as an ultrasonographic marker for monitoring tissue remodeling during follow-up. These findings suggest that ultrasonographic thickness and fibrillar organization may represent different dimensions of tissue remodeling. While fascial thickness remained relatively stable throughout follow-up, restoration of the fibrillar pattern was observed in most participants. This may indicate that qualitative architectural changes occur independently of measurable reductions in fascial thickness, supporting the use of fibrillar-pattern assessment as a complementary structural outcome.

### 4.1. Limitations

This study has several limitations that should be acknowledged. First, the assessment of the fibrillar pattern was performed qualitatively (recovery/no recovery), which may introduce a degree of imprecision depending on ultrasound image quality. Although inter-rater reliability analysis demonstrated almost perfect agreement between evaluators, and all images were independently reassessed by a second blinded examiner, the binary classification approach may still limit the precision of the structural analysis. Future studies should consider the development of more detailed semiquantitative classifications, including categories such as partial recovery and complete recovery of the fibrillar pattern, in order to provide a more comprehensive assessment of tissue remodeling.

Second, validated patient-reported outcome measures evaluating pain and function, such as the Visual Analog Scale (VAS), Foot Function Index (FFI), Foot and Ankle Disability Index (FADI), or Foot and Ankle Ability Measure (FAAM), were not systematically collected. Consequently, it was not possible to establish direct correlations between ultrasonographic structural changes and clinical evolution. Therefore, the findings of the present study should be interpreted strictly as structural imaging outcomes rather than evidence of clinical improvement or functional recovery.

Third, the presence of experimental losses—particularly associated with the intrafascial technique—may have influenced the estimation of the recovery rate. Nevertheless, this also provides relevant information regarding treatment tolerability. Specifically, two patients discontinued the protocol after the second treatment session because of perceived lack of benefit, while two additional patients withdrew due to severe pain associated with the first intrafascial injection. Future studies with controlled designs would help to strengthen the evidence obtained.

Another limitation is that the number of prolotherapy sessions was individualized according to patient evolution, which may have introduced a degree of treatment heterogeneity. Although all participants received the same injectate composition, ultrasound-guided technique, and standardized follow-up intervals, variability in the total number of infiltrations may have influenced the structural response observed during follow-up.

Finally, the heterogeneity of the sample in terms of disease duration may have influenced the response to treatment. Although the mean symptom duration of the sample was 13.39 ± 12.46 months, the minimum inclusion criterion of 30 days may limit comparability with studies defining chronic plantar fasciosis using longer symptom duration thresholds. Stratified analyses according to chronicity would therefore be of interest in future research.

Furthermore, restriction of the study population to individuals aged 30–45 years may limit the external validity of the findings, particularly in older populations commonly affected by plantar fasciosis.

In addition, although interobserver agreement for fibrillar pattern recovery was assessed using Cohen’s kappa coefficient, intraobserver reliability was not formally evaluated. Consequently, the reproducibility of repeated classifications by the same examiner could not be determined. Future studies should incorporate both interobserver and intraobserver reliability analyses to further strengthen the validity of ultrasound-based structural assessments.

### 4.2. Future Research Directions

The findings of the present study open new perspectives in the structural assessment of plantar fasciosis. First, future research should focus on validating the ultrasonographic fibrillar pattern as a marker of tissue remodeling through the development of semiquantitative or quantitative scales that allow for more objective and reproducible evaluation.

Furthermore, future investigations should incorporate validated clinical outcome measures, including pain and functional scales such as VAS, FFI, FADI, or FAAM, together with ultrasonographic assessment of fibrillar pattern recovery. This combined approach would help determine whether the observed structural remodeling is associated with clinically meaningful improvement and would allow validation of the fibrillar pattern as a biomarker of structural tissue remodeling and its relationship with symptomatic evolution. In this regard, studies integrating clinical, ultrasonographic, and biomechanical variables could provide a more comprehensive understanding of tissue remodeling and its relationship with symptomatic evolution.

Finally, it would be advisable to explore the evolution of the fibrillar pattern using advanced imaging techniques, such as elastography, as well as to assess long-term outcomes beyond one year of follow-up.

## 5. Conclusions

In patients with plantar fasciosis treated with ultrasound-guided 20% dextrose prolotherapy, recovery of the fibrillar pattern was observed in a high proportion of cases after one year of follow-up, whereas plantar aponeurosis thickness did not show significant longitudinal changes.

These findings suggest that assessment of the fibrillar pattern may provide additional information regarding structural tissue remodeling following prolotherapy, whereas plantar aponeurosis thickness remained relatively stable throughout follow-up. Consequently, the ultrasonographic fibrillar pattern may represent a useful marker for monitoring structural changes during follow-up and warrants further investigation in future studies.

The perifascial approach showed a higher rate of fibrillar pattern recovery than the intrafascial approach in this sample; however, these findings should be interpreted cautiously due to the limited sample size and the presence of treatment discontinuations in the intrafascial group. Further randomized studies with standardized ultrasound classification systems and clinical outcome correlation are warranted.

## Figures and Tables

**Table 1:** Baseline demographic, clinical, and ultrasound characteristics according to treatment group.

Variable	Intrafascial (n = 20)	Perifascial (n = 36)	*p*-Value
Female sex, n (%)	12 (60.0)	32 (88.9)	0.018 *
BMI (kg/m^2^)	27.65 ± 3.17	30.19 ± 5.29	0.029 *
Height (cm)	166.75 ± 9.41	165.94 ± 8.63	0.747
Symptom duration (months)	5.35 ± 4.83	9.11 ± 8.21	0.036 *
Baseline plantar aponeurosis thickness (cm)	0.639 ± 0.110	0.610 ± 0.114	0.365
Flat morphology, n (%)	14 (70.0)	24 (66.7)	1.000 *
Biconvex morphology, n (%)	6 (30.0)	12 (33.3)	1.000 *

Abbreviations: BMI = body mass index; cm = centimetres. Continuous variables are presented as mean ± standard deviation. Categorical variables are presented as absolute frequencies and percentages. Continuous variables were compared using independent-samples *t*-tests or Welch’s *t*-tests when homogeneity of variances was not met. Categorical variables were compared using Fisher’s exact test. Statistically significant between-group differences were observed for sex distribution, body mass index, and symptom duration. No significant between-group differences were observed for baseline plantar aponeurosis thickness or baseline plantar aponeurosis morphology. These latter variables represented the principal structural ultrasound characteristics evaluated in the present study. * = Statistically significant.

**Table 2:** Longitudinal changes in plantar aponeurosis thickness (cm) according to prolotherapy technique during the 52-week follow-up period.

Time Point	Global (Mean ± SD)	Intrafascial (Mean ± SD)	Perifascial (Mean ± SD)	*p*-Value (Time-Effect)
Baseline	0.620 ± 0.113	0.639 ± 0.110	0.610 ± 0.114	
2 weeks	0.620 ± 0.113	0.639 ± 0.110	0.610 ± 0.114	
4 weeks	0.620 ± 0.113	0.639 ± 0.110	0.610 ± 0.114	
6 weeks	0.624 ± 0.182	0.607 ± 0.177	0.634 ± 0.186	
8 weeks	0.589 ± 0.160	0.561 ± 0.221	0.605 ± 0.115	
12 weeks	0.562 ± 0.188	0.532 ± 0.251	0.579 ± 0.143	
26 weeks	0.558 ± 0.186	0.501 ± 0.275	0.591 ± 0.101	
52 weeks	0.556 ± 0.189	0.501 ± 0.275	0.586 ± 0.110	0.260

Abbreviations: cm = centimetres; SD = standard deviation; l. Repeated-measures GLM demonstrated no significant longitudinal changes in plantar aponeurosis thickness over time (Greenhouse–Geisser corrected: F = 1.365, *p* = 0.260, partial η^2^ = 0.027). No significant group × time interaction was observed between intrafascial and perifascial approaches (Greenhouse–Geisser corrected: F = 2.351, *p* = 0.093, partial η^2^ = 0.046). Mauchly’s test indicated violation of sphericity assumptions (W < 0.001); therefore, Greenhouse–Geisser correction was applied.

**Table 3:** Recovery of the fibrillar pattern at 52 weeks according to prolotherapy injection technique.

Group	Recovered, *n* (%)	Not Recovered, *n* (%)	*p*-Value
Intrafascial (*n* = 20)	16 (80.0)	4 (20.0)	
Perifascial (*n* = 36)	36 (100.0)	0 (0.0)	
Total (*n* = 56)	52 (92.9)	4 (7.1)	0.013

Abbreviations: n = number of participants. Fisher’s exact test comparing fibrillar pattern recovery between intrafascial and perifascial prolotherapy groups. Recovery of the fibrillar pattern was observed in 52 of 56 participants (92.9%). All non-recovered cases belonged to the intrafascial group. Fisher’s exact test demonstrated a statistically significant association between injection technique and fibrillar pattern recovery (*p* = 0.013).

**Table 4:** Interobserver agreement for fibrillar pattern recovery classification at 52 weeks.

Primary Examiner	Second Examiner: Recovery	Second Examiner: No Recovery	Total
Recovery	49	3	52
No recovery	0	4	4
Total	49	7	56
Cohen’s κ	0.83		

Abbreviations: κ = Cohen’s kappa coefficient. Agreement between the primary and second examiner was assessed using Cohen’s kappa coefficient. According to the classification proposed by Landis and Koch, a κ value of 0.83 indicates almost perfect interobserver agreement.

## Data Availability

The original contributions presented in the study are included in the article/, further inquiries can be directed to the corresponding author(s).
